# Drought-triggered leaf transcriptional responses disclose key molecular pathways underlying leaf water use efficiency in sugarcane (*Saccharum* spp.)

**DOI:** 10.3389/fpls.2023.1182461

**Published:** 2023-05-08

**Authors:** Danyel F. Contiliani, João Felipe C. de O. Nebó, Rafael V. Ribeiro, Marcos G. de A. Landell, Tiago C. Pereira, Ray Ming, Antonio Figueira, Silvana Creste

**Affiliations:** ^1^ Graduate Program in Genetics, Ribeirão Preto Medical School, Universidade de São Paulo, Ribeirão Preto, SP, Brazil; ^2^ Sugarcane Center, Agronomic Institute (IAC), Ribeirão Preto, SP, Brazil; ^3^ Centro de Energia Nuclear na Agricultura (CENA), Universidade de São Paulo, Piracicaba, SP, Brazil; ^4^ Department of Plant Biology, Institute of Biology, University of Campinas, Campinas, SP, Brazil; ^5^ Department of Biology, Faculty of Philosophy, Sciences, and Letters of Ribeirão Preto, Universidade de São Paulo, Ribeirao Preto, SP, Brazil; ^6^ Department of Plant Biology, University of Illinois Urbana-Champaign, Urbana, IL, United States

**Keywords:** abiotic stress, antioxidant mechanism, carboxylation efficiency, transcriptome, water use efficiency

## Abstract

Drought is a major constraint to sugarcane (*Saccharum* spp.) production and improving the water use efficiency (WUE) is a critical trait for the sustainability of this bioenergy crop. The molecular mechanism underlying WUE remains underexplored in sugarcane. Here, we investigated the drought-triggered physiological and transcriptional responses of two sugarcane cultivars contrasting for drought tolerance, ‘IACSP97-7065’ (sensitive) and ‘IACSP94-2094’ (tolerant). After 21 days without irrigation (DWI), only ‘IACSP94-2094’ exhibited superior WUE and instantaneous carboxylation efficiency, with the net CO_2_ assimilation being less impacted when compared with ‘IACSP97-7065’. RNA-seq of sugarcane leaves at 21 DWI revealed a total of 1,585 differentially expressed genes (DEGs) for both genotypes, among which ‘IACSP94-2094’ showed 617 (38.9%) exclusive transcripts (212 up- and 405 down-regulated). Functional enrichment analyses of these unique DEGs revealed several relevant biological processes, such as photosynthesis, transcription factors, signal transduction, solute transport, and redox homeostasis. The better drought-responsiveness of ‘IACSP94-2094’ suggested signaling cascades that foster transcriptional regulation of genes implicated in the Calvin cycle and transport of water and carbon dioxide, which are expected to support the high WUE and carboxylation efficiency observed for this genotype under water deficit. Moreover, the robust antioxidant system of the drought-tolerant genotype might serve as a molecular shield against the drought-associated overproduction of reactive oxygen species. This study provides relevant data that may be used to develop novel strategies for sugarcane breeding programs and to understand the genetic basis of drought tolerance and WUE improvement of sugarcane.

## Introduction

Climate change is a major threat to natural ecosystems and global agriculture in the upcoming years (Malhi et al., 2020). Long-lasting droughts endanger crop production, and it represents the most detrimental abiotic stress to plant growth and development (Lakshmanan and Robinson, 2013; Ferreira et al., 2017). Sugarcane (*Saccharum* spp.), a main feedstock for sugar and biofuel production, is strongly impacted during dry summer spells (de Andrade et al., 2016), particularly in marginal or suboptimal areas for cultivation (Contiliani et al., 2022). Sugarcane yields can decrease by up to 70% under water-limiting conditions (Gosal et al., 2009). Therefore, sugarcane breeding programs are gathering biotechnological resources to develop drought-tolerant genotypes.

Drought triggers tissue-specific molecular signaling in plants to mitigate the impacts of water deficit stress on plant growth and development (Zandalinas et al., 2022). Transcriptional regulation associated with abscisic acid (ABA)-dependent and -independent pathways results in several morphological and physiological changes, such as stomatal closure, antioxidant system activation, and photoassimilate remobilization, among others (Ferreira et al., 2017). Drought-triggered responses are genotype-dependent and can vary contrastingly among sugarcane cultivars (Rocha et al., 2007; Silva et al., 2007; Sales et al., 2013; Nawae et al., 2020; Contiliani et al., 2022). For instance, the drought-tolerant sugarcane genotype ‘IACSP94-2094’ displays an enhanced antioxidant system that protects leaf photochemical reactions, ensuring the recovery after drought stress, supporting plant growth after exposure to water deficit (Sales et al., 2013; Contiliani et al., 2022). Thus, genotype-comparative studies can help to pinpoint the underlying mechanisms of drought tolerance of stress-resilient genotypes.

Water use efficiency (WUE) is a complex trait connected to plant biomass yield (Gago et al., 2014), described as the ratio between carbon fixation (*i.e*., assimilated CO_2_) and water consumption (*i.e*., transpiration). Importantly, WUE is often seen as a parameter associated with the level of drought tolerance in plants (Zhengbin et al., 2011). However, the multigenic nature of WUE demands the prospection of related genes through large-scale -omic approaches. The transcriptional profile of bioenergy crops with contrasting WUE has been examined to pinpoint their differing strategies to cope with drought (Fan et al., 2015; Fracasso et al., 2016; Yuan et al., 2022). While global expression studies of drought-stressed sugarcane genotypes with contrasting WUE are scarce, WUE is a highly relevant trait for promoting sustainable biomass production by sugarcane in a scenario of climate change.

Here we conducted a thorough investigation of the drought-triggered responses in two sugarcane cultivars contrasting for drought tolerance: ‘IACSP97-7065’ (drought-sensitive) and ‘IACSP94-2094’ (drought-tolerant). Plants were subjected to controlled water deficit regimes under greenhouse conditions, and physiological parameters (including WUE) were estimated after 21 days without irrigation (DWI) and after the rehydration stage (recovery). Massive leaf transcriptome analyses were carried out at the maximum drought stress (21 DWI) using the RNA-seq (Illumina platform), and gene expression data were validated by quantitative reversed transcript amplification (RT-qPCR). Based on functional analyses, we report several biological pathways that were remarkably responsive in the drought-tolerant genotype. Our results provide new clues to unveil the intrinsic drought coping strategies in sugarcane, which may be useful for biotechnological applications and plant breeding programs.

## Methods

### Plant material

We analyzed two sugarcane (*Saccharum* spp.) cultivars contrasting for drought tolerance, ‘IACSP97-7065’ (drought-sensitive) and ‘IACSP94-2094’ (drought-tolerant), which were developed by the “Sugarcane Program” (Agronomic Institute, Sugarcane Center, Ribeirao Preto, SP, Brazil). The cultivars displayed differential growth and yield in drought-prone areas of Cerrado (the Brazilian “savanna”) (Machado et al., 2009; Oliveira, 2012). The plant material was sourced from our germplasm collection, requiring no licensing for this study. The 7-month-old plants were grown in large tanks (0.6 m^3^) filled with soil previously analyzed (**Supplementary Table S1**) and fertilized (30 kg N ha^-1^, 100 kg P ha^-1^, and 120 kg K ha^-1^) according to Spironello et al. (1997), in a greenhouse and evaluated under water deficit (drought) or irrigated (control) conditions. Plants from both cultivars were cultivated together in six tanks – three irrigated (control) and three non-irrigated (drought) – in order to ensure that they were exposed to the same condition (Verslues et al., 2006). Each tank comprised one biological replicate of each cultivar. The (irrigated) control tanks were manually watered to field capacity. The water deficit treatment was imposed by water withholding and monitored by evaluating leaf water potential and photochemical and gas-exchange parameters (see below) until photosynthesis ceased – at 21 days without irrigation (DWI). Subsequently, all tanks were re-watered daily for plant recovery for seven days. The mean air and soil temperatures were 19.7°C and 22.9°C, respectively at 06:00 AM. The experiments reported herein comply with all relevant institutional and national guidelines.

For the transcriptome analysis, +1 leaves (the first fully expanded leaf) from both genotypes were collected at 21 DWI for RNA extraction. The experiments were conducted in biological triplicates (*n* = 3). Each biological replicate was composed of a bulk of three fully expanded and light-exposed (+1) leaves.

### Physiological analyses

The physiological responses of both sugarcane cultivars to water deficit were estimated at 21 DWI and rehydration stage (seven days after rewatering) compared to irrigated controls. An infrared gas analyzer (LI-6400XT, LI-COR; Lincoln, NE, USA) was used to measure chlorophyll fluorescence and leaf gas-exchange parameters, such as CO_2_ assimilation (P_N_), instantaneous carboxylation efficiency (CE_i_, given by the ratio between P_N_ and intercellular CO_2_ concentration), stomatal conductance (g_s_), transpiration (E), and water use efficiency (WUE, given by P_N_/E). These estimates were performed on +1 leaves under photosynthetic photon flux density of 2000 µmol m^-2^ s^-1^ and 385 ± 6 ppm CO_2_ concentration, considering natural air temperature (21 DWI – 29.3 to 31.9°C; rehydration - 24.3 to 28.0°C) and humidity (21 DWI – 22.7 to 34.0%; rehydration – 53.1 to 60.7%) between 09:00 and 11:00 AM. Some additional photochemical parameters were estimated, such as the maximum (F_v_/F_m_) and effective (Φ_PSII_) quantum efficiency of photosystem II (PSII) and the non-photochemical quenching (NPQ). F_v_/F_m_ is based on fluorescence signals from dark-adapted leaves, whereas Φ_PSII_ is based on fluorescence signals from light-adapted leaves, following the pulse saturation method (λ < 710 nm, Q ~ 12,000 µmol m^−2^ s^−1^, 0.8 s) (Schreiber, 2004). The leaf water potential (Ψ, MPa) was measured until 21 DWI using a Scholander pressure chamber (3005 model, Soilmoisture Equipment Co.; Goleta, CA, USA) at 06:00 AM. Technical and biological replicates were subjected to the three-way analysis of variance (ANOVA) considering genotype, treatment (21 DWI *vs.* rehydration), and condition factors (irrigated *vs.* drought), followed by Tukey’s test (*p*-value < 0.05). The physiological raw data are shown in **Supplementary Table S2**.

### RNA extraction and sequencing

Total RNA was extracted using a Lithium Chloride (LiCl) method (Leal et al., 2007) and purified by Ambion DNase I kit (Invitrogen; Waltham, MA, USA) according to the manufacturer’s recommendations. Total RNA was quantified using a Nanodrop 2000 spectrophotometer (Thermo Fisher Scientific; Waltham, MA, USA), and the integrity of the isolated RNA was assessed *via* 1.2% agarose electrophoresis. Libraries were assembled according to the recommendations of TruSeq RNA Sample Prep kit (Illumina; San Diego, CA, USA). Then, four cDNA libraries were paired-end sequenced by a HiSeq 2000 Illumina sequencer. CLC Genomics Workbench (QIAGEN, Hilden, Germany) software was used for quality check, filtering, and trimming (limit = 0.05, maximum number of ambiguities = 2) procedures. The raw transcriptome data were deposited in the NCBI Sequence Read Archive (SRA) database (BioProject ID: PRJNA882367).

### Differential gene expression analysis

Transcriptome mapping, gene count, and gene expression analysis were conducted by CLC Genomics Workbench software under default settings (length fraction = 0.5 and similarity fraction 0.8). Cleaned reads were mapped to Sugarcane Assembled Sequences (SAS) from Sugarcane EST Project (SUCEST) database (Vettore et al., 2001) as references, which comprise 43,141 contigs. Tablet software (https://ics.hutton.ac.uk/tablet/) was used to identify mismatches between reads and reference. Reads per kilobase of exon per million mapped reads (RPKM) were calculated, and differentially expressed genes (DEGs) were identified by CLC Genomics Workbench software contrasting drought versus irrigated treatments at *p*-value ≤ 0.05 and Benjamini and Hochberg adjusted FDR (*q*-value) < 0.05. Gene expression values were then normalized in log_2_Fold-change (FC). In this study, only DEGs with |log_2_FC| greater or equal to 1 were considered for further analyses.

### Functional annotation

The search for orthologs of the DEGs was executed by the BLASTX tool (*e*-value < 10^-5^) in OmicsBox v.1.1.164 (Götz et al., 2008; BioBam Bioinformatics, 2019) against the non-redundant protein databases of monocot plant species. Gene ontology (GO) terms were predicted based on Gene Ontology Annotation (GOA) database and analyzed *via* the WEGO tool (https://biodb.swu.edu.cn/cgi-bin/wego/index.pl), which determined the significance level (*p*-value < 0.05) between the gene numbers (related to GO terms) in the two sugarcane data sets (drought-tolerant versus drought-sensitive). Kyoto Encyclopedia of Genes and Genomes (KEGG) (Kanehisa and Goto, 2000; Kanehisa, 2019; Kanehisa et al., 2021) pathway annotation was performed using the KOBAS-i tool (Bu et al., 2021) against the *Sorghum bicolor* database (Benjamini-Hochberg adjusted *p*-value ≤ 0.05). To visualize the deregulated biological pathways, DEGs were first mapped to plant protein categories (BIN ontologies) from Mercator4 v5.0 (Lohse et al., 2014), then submitted to MapMan v.4 (Schwacke et al., 2019), which generated several diagrams for up- and down-regulated pathways (Benjamini-Hochberg adjusted *p*-value ≤ 0.05).

### Gene expression validation

Fifteen DEGs detected by RNA-Seq were randomly selected for gene expression validation by reversed transcription-quantitative polymerase chain reaction (RT-qPCR) analysis. Primer pairs are shown in **Supplementary Table S3**. The cDNA synthesis was performed using the RevertAid Premium Reverse Transcriptase (Thermo Fisher Scientific). The RT-qPCR assay was carried out in CFX96 Touch Real-Time PCR (Bio-Rad; Hercules, CA, USA) using 15 µL-reaction containing 7.5 µL Platinum SYBR Green qPCR SuperMix-UDG (Invitrogen), 0.3 µM of each primer, and 1 µL cDNA 1:10 (v:v). Amplification conditions were set at 95°C for 10 min, followed by 40 cycles of 95°C for 15 s, 60°C for 20 s, and 72°C for 20 s. Melting curve analysis between 72 and 95°C was conducted to confirm the specificity of the reaction. Three biological and technical replicates were used for each sample. Reference genes *25S rRNA1* and *25S rRNA2* were used as reference genes. RT-qPCR expression data was retrieved by Bio-Rad CFX Manager software, and statistical analysis (drought versus irrigated) was performed using the software REST 2009 (Pfaffl et al., 2002) with a *p*-value ≤ 0.05. Relative expression values between drought and irrigated treatments were determined using the comparative C_Q_ method (Schmittgen and Livak, 2008), followed by log_2_ normalization. Finally, the reliability of the RNA-Seq data was assessed by Spearman’s rank correlation test (*p*-value ≤ 0.05) using the gene expression values from the RNA-Seq and RT-qPCR experiments. This correlation analysis was performed using GraphPad Prism v. 8.0.0 for Windows (GraphPad Software, San Diego, CA, USA; www.graphpad.com).

## Results

### Physiological responses of drought-stressed and recovered sugarcane plants

**Figure 1:**
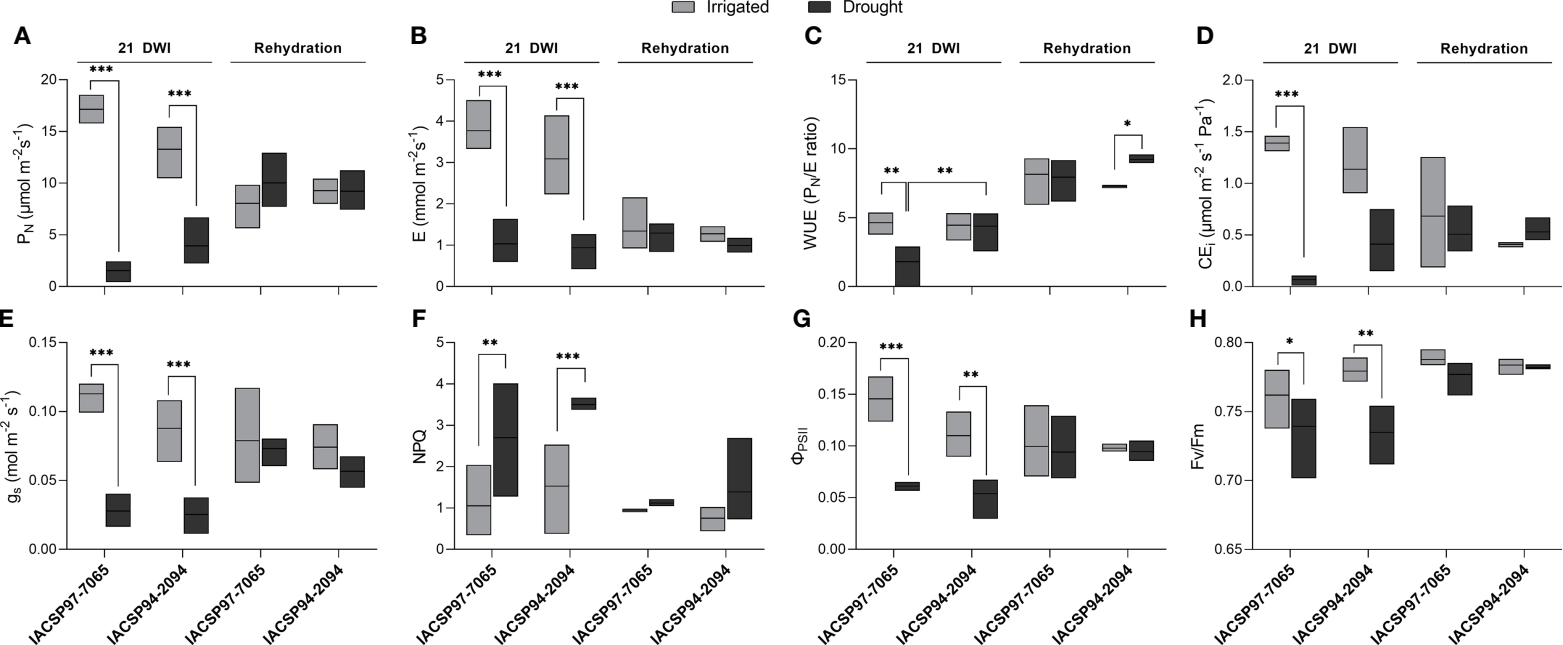
Physiological changes in drought-stressed sugarcane genotypes. Drought-sensitive (‘IACSP97-7065’) and -tolerant (‘IACSP94-2094’) sugarcane genotypes were physiologically evaluated at 21 days without water (21 DWI) and rehydration (recovery) for irrigated (control) and drought groups. The physiological parameters examined were: **(A)** CO_2_ assimilation (P_N_), **(B)** transpiration **(E)**, **(C)** water use efficiency (WUE, P_N_/E), **(D)** instantaneous carboxylation efficiency (CE_i_), **(E)** stomatal conductance (g_s_), **(F)** non-photochemical quenching (NPQ), **(G)** maximum (F_v_/F_m_) and **(H)** effective (Φ_PSII_) quantum efficiency of photosystem II. Data represent the means ± SE (*n* = 3 biological replicates) and statistical differences are represented as *P<0.05, **P<0.01, and ***P<0.001.

Changes in physiological parameters of both cultivars were investigated at the maximum water deficit (21 DWI) and after rehydration (**Figure 1**). Leaf water potential measurements throughout the experiment indicated that both ‘IACSP97-7065’ and ‘IACSP94-2094’ plants were under severe water deficit at 21 DWI (**Supplementary Figure S1**). In consequence, a substantial decrease in net CO_2_ assimilation was observed for both the drought-tolerant (70%) and -sensitive (91%) cultivars (**Figure 1A**). Plants from both genotypes exposed to water deficit showed significant changes in the physiological parameters evaluated, such as transpiration rate (**Figure 1B**), stomatal conductance (**Figure 1E**), non-photochemical quenching (**Figure 1F**), and effective (**Figure 1G**) and maximum (**Figure 1H**) quantum efficiency of photosystem II. Taken together, the data indicate that non-irrigated plants from both cultivars were facing a water deficit at 21 DWI. However, only plants of ‘IACSP94-2094’ under water deficit were able to maintain their WUE (**Figure 1C**) and the instantaneous carboxylation efficiency (CE_i_; **Figure 1D**) at 21 DWI, whereas the drought-sensitive genotype showed a significant decrease in WUE (-61%) and CE_i_ (-95%).

All the gas-exchange and photochemical parameters were recovered after rehydration of the plants from both cultivars and all plants showed similar values, but at lower levels than those observed for the irrigated control plants. Notably, the WUE of the rehydrated ‘IACSP94-2094’ plants remained at higher levels in comparison to the control (**Figure 1C**).

### Transcriptome of drought-stressed sugarcane leaves and its validation

Paired-end RNA sequencing provided a total of 133,059,140 reads with 100 base pairs from the four libraries involving both cultivars (‘IACSP97-7065’ and ‘IACSP94-2094’) and watering regime treatments, which rendered a total of 113,815,386 (85%) high-quality paired-end reads after quality control (**Supplementary Table S4**). Leaf transcriptome mapping against the Sugarcane Assembly Sequences (SAS) revealed 74,409,560 uniquely mapped transcripts with an average length of 828 base pairs. Gene expression analyses revealed a normal distribution in the number of reads mapped for each gene/contig (gene counts) transformed to log scale (**Supplementary Figure S2**). Therefore, RPKM values were determined based on those uniquely mapped reads for drought or irrigated (control) plants.

**Figure 2:**
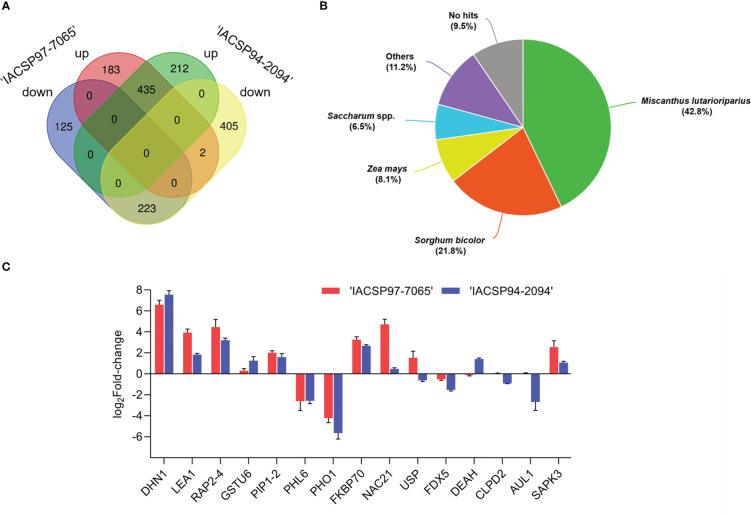
RNA-Seq results and RT-qPCR validation. **(A)** the Venn diagram represents the distribution of exclusive and shared up- and down-regulated DEGs between sugarcane genotypes. **(B)** Overall annotation of leaf sugarcane DEGs against monocots protein databases. **(C)** Validation of the expression profiles of 15 selected genes from ‘IACSP97-7065’ (red) and ‘IACSP94-2094’ (blue) at 21 DWI, in which relative expression levels (drought *vs*. irrigated) are represented as log_2_Fold-change. Error bars represent standard deviations.

This study revealed a total of 1,585 (3.6%) differentially expressed genes (**Supplementary Table S5**) based on log-transformed RPKM values (*p*-value ≤ 0.05). From these, ‘IACSP97-7065’ displayed 308 particular DEGs (183 up- and 125 down-regulated), whereas ‘IACSP94-2094’ exhibited 617 particular DEGs (212 up- and 405 down-regulated). Moreover, the sugarcane genotypes shared numerous similarly regulated genes (435 up- and 223 down-regulated) and only 2 with opposite transcriptional profiles (**Figure 2A**). Functional annotation resulted in 1,450 (91.5%) well-annotated transcripts (**Figure 2B**) against *Saccharum* spp. sequences (6.5%) and to close grass species, such as *Miscanthus lutarioriparius* (42.8%), *Sorghum bicolor* (21.8%), *Zea mays* (8.1%), and others (11.2%). The top BLAST hits for each annotated DEG are listed in **Supplementary Table S6**.

The expression levels of 15 randomly chosen DEGs obtained by RNA-seq were validated by RT-qPCR analysis. The differential expression of 14 genes between the leaf samples from plants of both sugarcane genotypes upon drought treatment from the RNA-seq data was corroborated by the RT-qPCR analysis (**Figure 2C**), with only one gene (*chaperone protein*, *CLPD2*) showing no differential expression. The reproducibility and accuracy of the expression levels of the 15 DEGs were supported by a strong correlation (Spearman’s *r* ^2 = ^0.85; *p*-value < 0.0001) between the RNA-seq and RT-qPCR data (**Supplementary Figure S3**).

Considering the RT-qPCR data (**Figure 2C**), the validated genes displayed similar transcriptional profiles (up- or down-regulation) between genotypes, but with distinct intensities. For instance, *late embryogenesis abundant-1* (*LEA1*), a protein involved in tolerance to extreme desiccation (Pantelić et al., 2022) was shown to be 2-fold more induced in ‘IACSP97-7065’ compared to the drought-tolerant genotype at 21 DWI in relation to watered control plants. Likewise, *serine/threonine-protein kinase* (*SAPK3*) was 2.3-fold more expressed in plants of the drought-sensitive genotype as compared to those from ‘IACSP94-2094’. The fact that some genes showed differential expression for only one of the genotypes is noteworthy. The *NAC21* transcription factor (log_2_FC = 4.7) and *universal stress protein* (*USP*; log_2_FC = 1.54) were up-regulated, particularly in ‘IACSP97-7065’, whereas the chloroplast *ferredoxin-5* was down-regulated (log_2_FC = -1.53). On the other hand, the ‘IACSP94-2094’ genotype exhibited exclusive differential expression for *ATP-dependent RNA helicase A-like* (*DEAH*; log_2_FC = 1.42), *auxin-like 1* (*AUL1*; log_2_FC = -2.67), and *tau class glutathione S-transferase 6* (*GSTU6*; log_2_FC = 1.2).

### Functional annotation of the sugarcane DEGs

**Figure 3:**
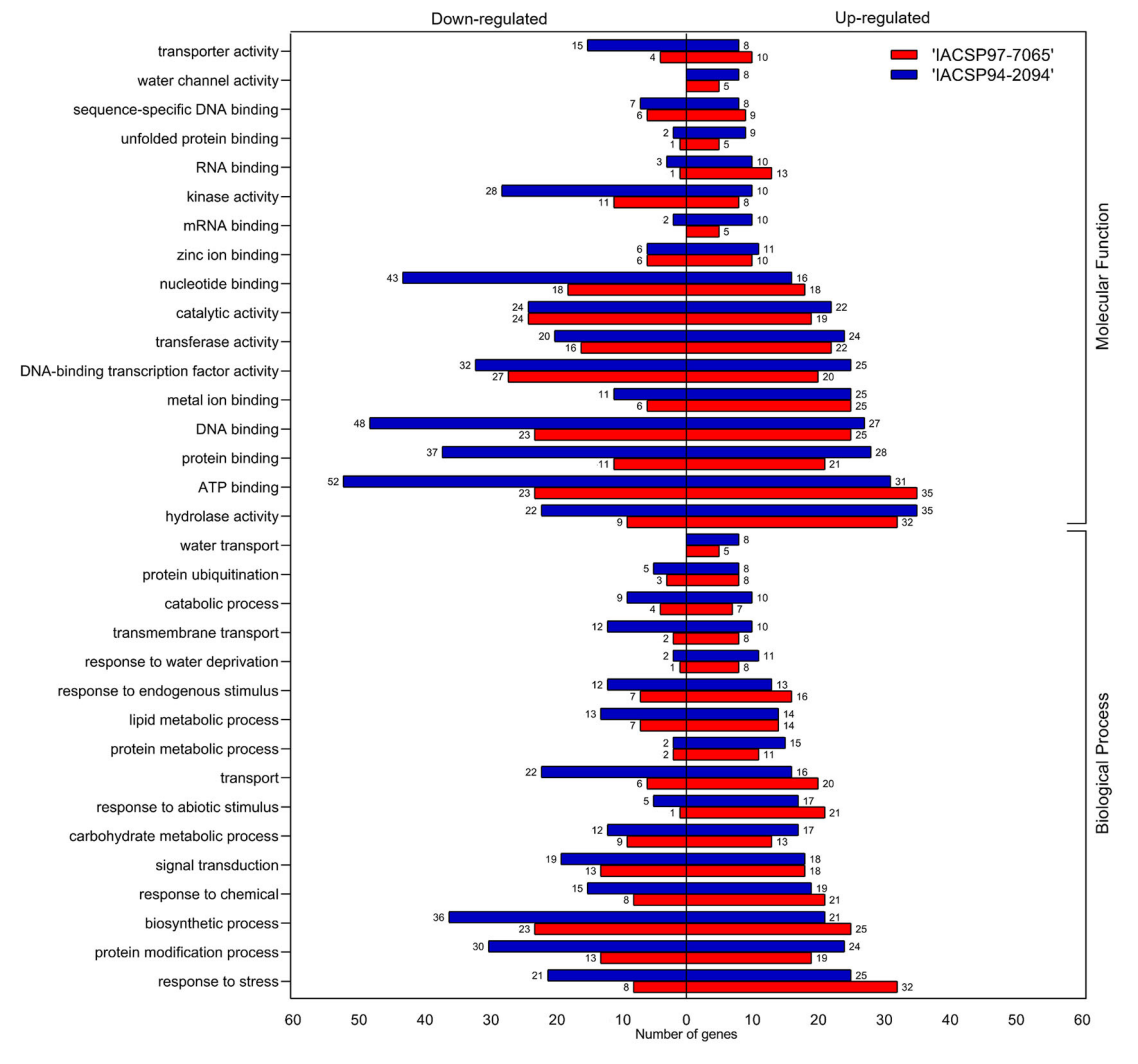
Gene ontology (GO) functional classification of sugarcane DEGs. GO terms are divided into ‘molecular function’ and ‘biological process’ categories, which are represented by up- (right) and down-regulated (left) genes from ‘IACSP97-7065’ (red) and ‘IACSP94-2094’ (blue) sugarcane genotypes.

The Gene Ontology (GO) enrichment analysis highlighted many GO terms for the transcriptomic analysis of the plants from both cultivars under drought stress (**Figure 3**). Categories of genes annotated as ‘molecular functions’ and ‘biological processes’ were separated as down- or up-regulated. ‘IACSP94-2094’ showed a higher number of overall DEGs (up- and down-regulated) associated with most GO terms. ‘IACSP94-2094’ displayed several up-regulated DEGs categorized into some GO terms, such as ‘protein binding’ (28 members), ‘DNA-binding transcription factor activity’ (25), ‘carbohydrate metabolic process’ (17), ‘protein metabolic process’ (15), ‘response to water deprivation’ (11), ‘unfolded protein binding’ (9), ‘water transport’ (8), and ‘water channel activity’ (8). The most contrasting GO terms with repressed genes in ‘IACSP94-2094’were ‘ATP binding’ (52), ‘DNA binding’ (48), ‘nucleotide binding’ (43), ‘protein binding’ (37), ‘biosynthetic process’ (36), ‘protein modification process’ (30), ‘kinase activity’ (28), ‘hydrolase activity’ (22), ‘transport’ (22), ‘response to stress’ (21), ‘transporter activity’ (15), ‘lipid metabolic process’ (13), ‘transmembrane transport’ (12), and ‘catabolic process’ (9). Conversely, GO terms were less represented for the ‘IACSP97-7065’ samples but revealed a higher number of up-regulated DEGs in some terms, such as ‘ATP binding’ (35), ‘response to stress’ (32), ‘biosynthetic process’ (25), ‘response to abiotic stimulus’ (21), ‘transport’ (20), ‘nucleotide binding’ (18), ‘response to endogenous stimulus’ (16), ‘RNA binding’ (13), and ‘transporter activity’ (10).

**Figure 4:**
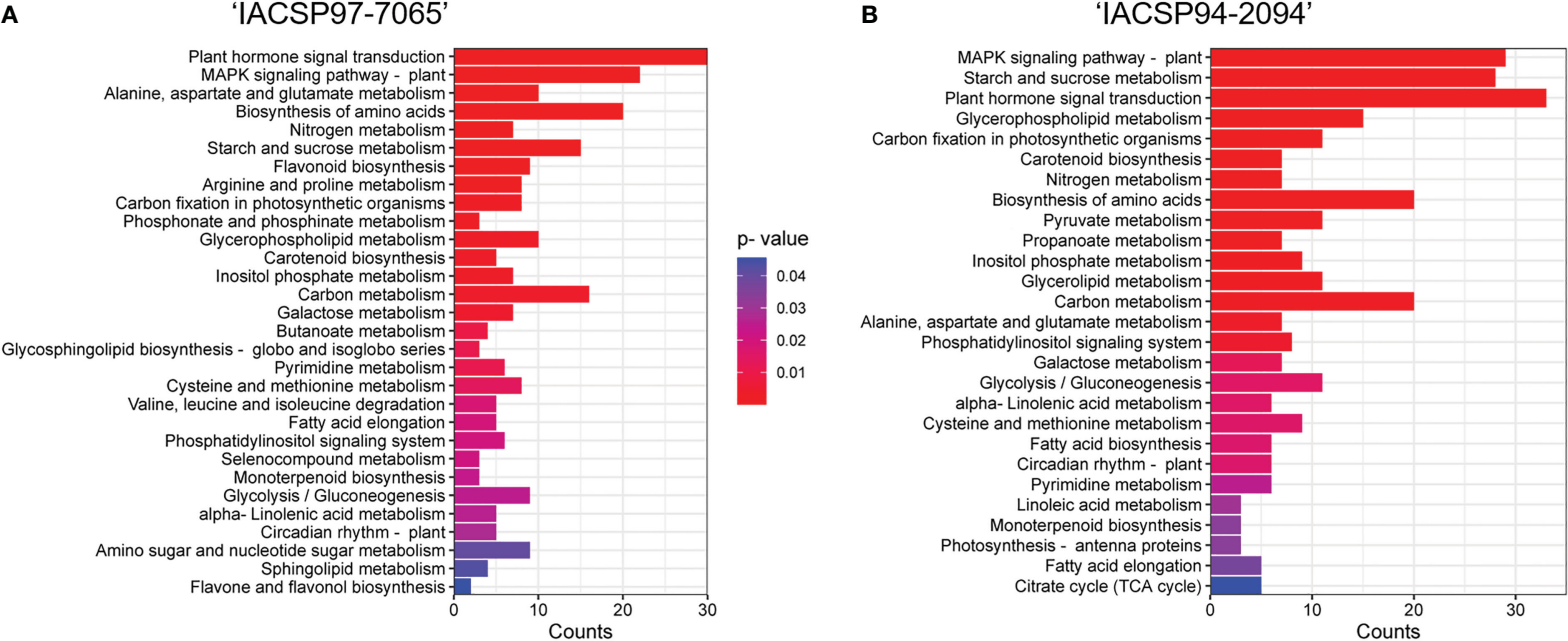
Enriched KEGG pathway categories. KOBAS analysis of sugarcane DEGs enriched the responsive biological pathways for drought-stressed **(A)** ‘IACSP97-7065’ and **(B)** ‘IACSP94-2094’ genotypes. Gene counts for each pathway are represented by the X-axis. The significantly enriched pathways represent their corrected *p*-values according to the color spectrum (from red to blue).

KEGG pathway enrichment analysis indicated additional ‘IACSP94-2094’ and ‘IACSP97-7065’ DEGs involved in plant hormone signal transduction, MAPK signaling pathway, starch and sucrose metabolism, biosynthesis of amino acids, carbon fixation in photosynthetic organisms, carbon metabolism, glycerophospholipid metabolism, among others (**Figure 4**). While ‘IACSP94-2094’ showed more genes represented in most of the enriched pathways (**Figure 4B**), it also displayed exclusive pathways, such as pyruvate metabolism, propanoate metabolism, glycerolipid metabolism, fatty acid biosynthesis, linoleic metabolism, photosynthesis – antenna proteins, and citrate cycle (TCA cycle). It is noteworthy that the drought-tolerant ‘IACSP94-2094’ showed a higher number of genes associated with carbon fixation in photosynthetic organisms and carbon metabolism, along with photosynthesis – antenna proteins. Conversely, the sample of ‘IACSP97-7065’ showed some particular enriched pathways (**Figure 4A**), such as flavonoid metabolism, arginine and proline metabolism, phosphonate and phosphinate metabolism, butanoate metabolism, glycosphingolipid biosynthesis – globo and isoglobo series, valine, leucine and isoleucine degradation, selenocompound metabolism, amino sugar and nucleotide sugar metabolism, sphingolipid metabolism, and flavone and flavonol metabolism.

**Figure 5:**
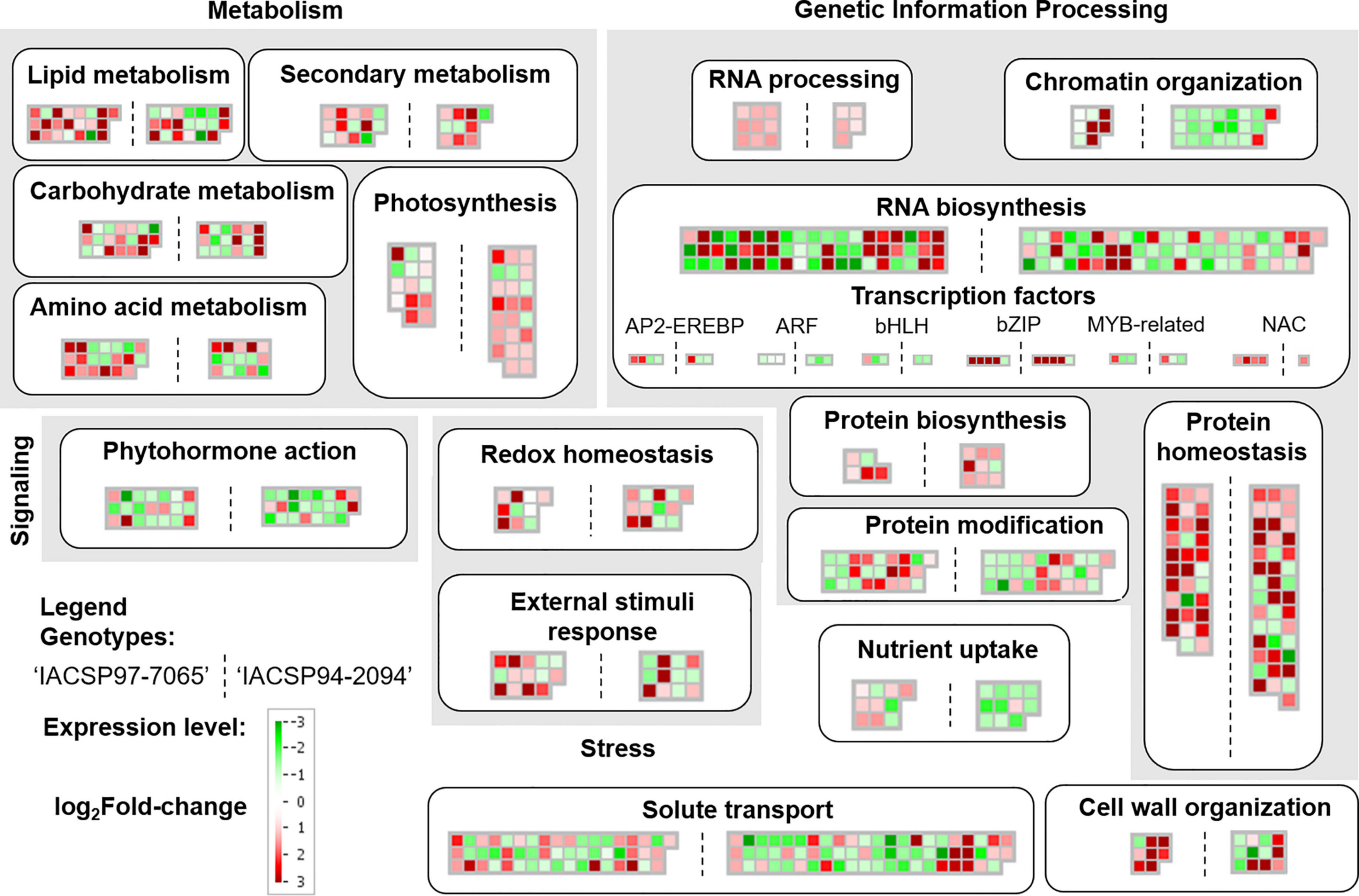
Mapman annotation of gene expression profiles. Functional classes (indicated in bold within boxes) comprise transcriptional profiles of ‘IACSP97-7065’ (left) and ‘IACSP94-2094’ (right) sugarcane genotypes. Each mapped gene is displayed in square within heatmaps, and the color scale indicates its relative expression (log_2_Fold-change). Up-regulated DEGs are represented in red, whereas down-regulated ones are represented in green.

Furthermore, MapMan analysis revealed other sets of differentially expressed biological pathways in leaves of both sugarcane genotypes coping with water deficit (**Figure 5**). These pathways were related to the metabolism of amino acids, secondary metabolism, redox homeostasis, external stimuli response, RNA processing and biosynthesis, chromatin organization, nutrient uptake, cell wall organization, among others (**Figure 5**). Notably, the drought-tolerant genotype showed a broader set of mapped genes mainly up-regulated in several pathways, including photosynthesis, chromatin organization, solute transport, cell wall organization, redox homeostasis, and RNA biosynthesis. Conversely, the drought-sensitive genotype showed DEGs more representative of metabolism of amino acids, RNA processing, and transcription factors (*AP2-EREBP*, *bHLH*, and *NAC*) (**Figure 5**).

### Dissecting the DEGs of drought-stressed sugarcane leaves

**Figure 6:**
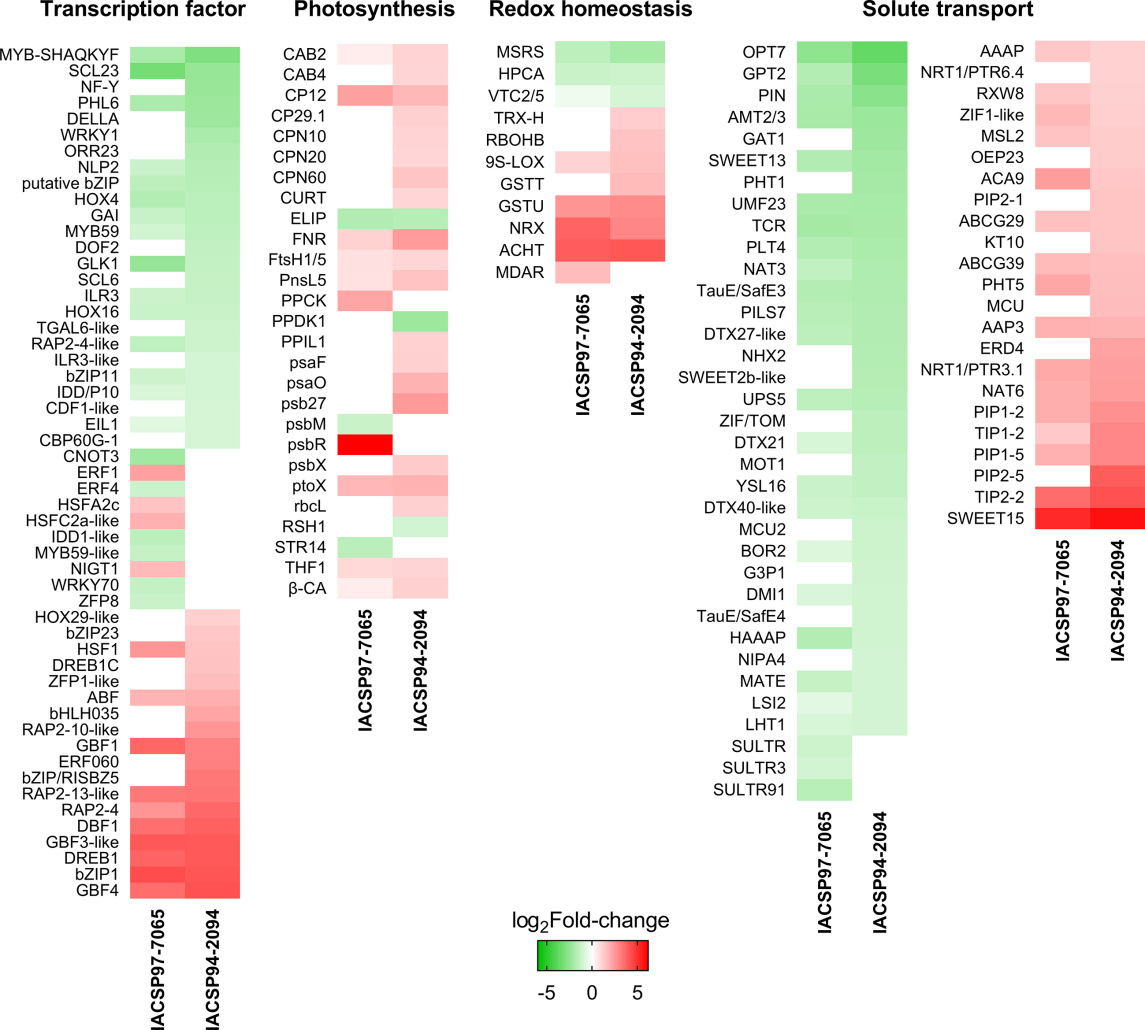
Expression-based heatmaps of drought-responsive sugarcane genes. Transcriptional profiles of sugarcane genotypes are represented in four functional categories: transcription factor, photosynthesis, redox homeostasis, and solute transport. Color scale indicates relative gene expression (log_2_Fold-change), ranging from down- (green) to up-regulated (red). Non-significant gene expression is colored in white.

Several DEGs are classified in the drought-responsive biological pathways that were functionally predicted for both genotypes. To identify these genes, we dissected the gene expression of DEGs associated with (i) transcription factors, (ii) photosynthesis, (iii) redox homeostasis, and (iv) solute transport (**Figure 6**). The sugarcane genotypes share many DEGs within these four classes; yet most of these were found exclusively in the more tolerant ‘IACSP94-2094’ plants.

Focusing on the drought-tolerant genotype, the set of transcription factors comprised 18 DEGs (10 down- and eight up-regulated) not found in ‘IACSP97-7065’, including *NF-Y* (SCRLLR1109E12.g), *DELLA* (SCQSST1037G07.g), *WRKY1* (SCQGLR2025A07.g), *ORR23* (SCJFSB1011E04.g), *DOF2* (SCRLAD1136C12.g), *SCL6* (SCBFRZ2016F05.g), *TGAL6-like* (SCRURT2012D03.g), *ILR3-like* (SCCCRT1003E06.g), *CDF1-like* (SCRLAD1136C12.g), and *CBP60G-1* (SCEQRT2025A05.g) as down-regulated, and *HOX29-like* (SCCCAM1C09G11.g), *bZIP23* (SCVPLB1018G11.g), *DREB1C* (SCQGLR1041E11.g), *ZFP1-like* (SCMCLR1122C01.g), *bHLH035* (SCEZRZ1016C11.g), *RAP2-10-like* (SCRLAD1043B06.g), *ERF060* (SCRLLV1050E06.g), and *bZIP/RISBZ5* (SCCCCL4005C09.g) as up-regulated (**Figure 6**). The ‘photosynthesis’ set highlighted the overall positive responsiveness of ‘IACSP94-2094’ with 14 exclusive DEGs (2 down- and 12 up-regulated), such as *PPDK1* (SCRLCL6031H05.g) and *RSH1* (SCEZRZ3051A09.g) as down-regulated and *CAB4* (SCCCST1002F06.g), *CP29.1* (SCUTST3131A12.g), *CPN10* (SCUTLR1058D06.G), *CPN20* (SCJLLR1106F03.G), *CPN60* (SCCCCL4005E12.G), *CURT* (SCMCSD1061A04.G), *PPIL1* (SCEQFL5044B07.G), *psaF* (SCACSB1039A11.G), *psaO* (SCJFAD1013H02.G), *psb27* (SCUTSB1076D12.G), *psbX* (SCRFLB1055B09.G), and *rbcL* (SCJFLR1073E09.g) as up-regulated (**Figure 6**). The redox homeostasis set indicated only three up-regulated DEGs found exclusively in ‘IACSP94-2094’, including *TRX-H* (SCAGLR2011H07.G), *RBOHB* (SCJLRT1023G05.G), and *GSTT* (SCBGLR1002F10.G) (**Figure 6**). Finally, the solute transport set was the most represented set for both genotypes, with the ‘IACSP94-2094’ counting 17 DEGs (10 down- and seven up-regulated), including *GAT1* (SCEZRZ3015D04.G), *PHT1* (SCCCLR1001G10.G), *NHX2* (SCRLFL4028D02.G), *SWEET2b-like* (SCBGLR1120E06.G), *ZIF/TOM* (SCCCCL7002B06.G), *MOT1* (SCJLRT2052C07.G), *MCU2* (SCQSLB1049D10.G), *G3P1* (SCCCST2002F04.G), *TauE/SafE4* (SCCCLR1C03H03.G), and *NIPA4* (SCJLST1020D04.G) as down-regulated, and *NRT1/PTR6.4* (SCACRZ3035B05.G), *OEP23* (SCCCST3C03A11.G), *PIP2-1* (SCRFLR1012A08.G), *KT10* (SCCCST2002G11.G), *MCU* (SCBFRT1064C07.G), *ERD4* (SCJLRZ1024A10.G), and *PIP2-5* (SCJFRT1059C11.G) as up-regulated. Within these four sets (transcription factors, photosynthesis, redox homeostasis, and solute transport), the drought-tolerant genotype highlights its greater drought-responsiveness with 52 exclusive DEGs, whereas the drought-sensitive showed only 18.

## Discussion

Drought triggers a broad spectrum of physiological, biochemical, and molecular responses across sugarcane cultivars (Rocha et al., 2007; Silva et al., 2007; Sales et al., 2013; Ferreira et al., 2017; Ngara et al., 2021). Typically, sugarcane plants exposed to water deficit are prone to exhibit a decrease in photosynthetic rate due to stomatal and non-stomatal (*e.g.*, metabolic impairment) limitations, as well as decreases in transpiration rate (Du et al., 1996; Inman-Bamber and Smith, 2005; Graça et al., 2010; Medeiros et al., 2013; Basnayake et al., 2015). This study investigated the physiological responses of two sugarcane cultivars, a drought-sensitive (‘IACSP97-7065’) and drought-tolerant (‘IACSP94-2094’) (Sales et al., 2013; Contiliani et al., 2022), at 21 days without irrigation (DWI) and rehydration. Leaf CO_2_ assimilation, stomatal conductance, transpiration, the maximum and effective quantum efficiencies of PSII) were reduced for both cultivars at 21 DWI (**Figures 1A, B, E-H**), indicating that these plants were indeed under water deficit stress. A reduced PSII quantum efficiency has been considered a suitable indicator of drought-stressed sugarcane plants (Kim et al., 2006). However, WUE was found exceptionally maintained in ‘IACSP94-2094’ plants, whereas ‘IACSP97-7065’ displayed a significant decrease compared to the irrigated control. Carboxylation efficiency (CE_i_) was also maintained in ‘IACSP94-2094’ plants at 21 DWI, whereas a substantial decrease was found in ‘IACSP97-7065’, which suggests a biochemical impairment of photosynthesis in the drought-sensitive genotype. Upon rehydration, WUE was significantly higher in ‘IACSP94-2094’ compared to its irrigated control. The improvement of WUE in ‘IACSP94-2094’ may be explained by the apparent slow recovery of stomatal conductance (**Figure 1E**, *p-*value = 0.063), as severe drought stress could accumulate high concentrations of ABA that might take longer to be degraded (Miranda et al., 2022). Together, these results show the ability of the tolerant genotype to maintain CO_2_ assimilation with reduced transpiration – improved WUE – under unfavorable conditions, a strategy that can save water and benefit field-grown plants in the long term. Consistently to other reports (Sales et al., 2013; Contiliani et al., 2022), our physiological data support the drought tolerance of the ‘IACSP94-2094’ cultivar.

Intricate genetic networks are spatiotemporally fine-tuned to regulate proper physiological responses to drought stress in a genotype-dependent manner (Ngara et al., 2021). This transcriptome study relied on the RNA-seq approach to unveil the differential transcriptional adaptations of ‘IACSP94-2094’ and ‘IACSP97-7065’ upon water deficit conditions. Our findings revealed a higher number of responsive transcripts in the water-limited condition for the drought-tolerant genotype compared to the sensitive one, similar to a microarray-based study conducted in a drought-prone sugarcane field (Contiliani et al., 2022). Furthermore, functional annotation of sugarcane DEGs revealed that ‘IACSP94-2094’ and ‘IACSP97-7065’ exhibited transcriptional responses comprising a wide range of biological pathways, in which they have different associated genes, thus reinforcing their differing molecular strategies to cope with drought.

During water deficit, plants strive to adapt to impending ion imbalances in the drying soil by controlling ion flow into the cells. Ion homeostasis is regulated by primary active (*e.g*., H^+^-ATPases) and secondary (*e.g*., co-transporters and membrane channels) transport (Conde et al., 2011). Despite the large number of repressed transport-related genes in both sugarcane genotypes, our data showed that ‘IACSP94-2094’ displayed up-regulated DEGs involved in solute transport, including plasma intrinsic membrane proteins (*PIP2-1* and *PIP2-5*), K^+^ transporter (*KT10*), chloroplast outer envelope protein (*OEP23*), mitochondrial calcium uptake channel (*MCU*), and early-responsive to dehydration 4 (*ERD4*). In hexaploid wheat, *OEP16-2* is responsive to heat stress and confers drought tolerance when overexpressed in *Arabidopsis thaliana* transgenic lines (Zang et al., 2017). Similarly, *ERD4* was reported as up-regulated in drought-stressed sugarcane (Devi et al., 2019) and maize plants (Liu et al., 2009) and promotes tolerance to water deficit and high salinity when overexpressed in *A. thaliana* plants (Liu et al., 2009). PIP aquaporins facilitate the transport of water and CO_2_ across cell membranes (Maurel et al., 2008), and have been reported in sugarcane transcriptional analyses as highly responsive to abiotic stresses, including drought (de Andrade et al., 2016) and salinity (Tang et al., 2021). Considering that *PIP*-overexpressing plants displayed improved tolerance to drought and salt stress, and enhanced WUE (Sade et al., 2010; Li et al., 2016; Wang et al., 2017), the up-regulation of *PIP2-1* and *PIP2-5* in ‘IACSP94-2094’ under water deficit suggest these aquaporins might have a role for the observed enhanced WUE and drought tolerance of this cultivar. As aquaporins might facilitate the internal transport of water, further studies should investigate the role of these proteins on water distribution in leaf mesophyll.

Plant signal transduction to drought stress often involves ABA-dependent mitogen-activated protein kinase (MAPK) cascade to elicit a physiological response (Lin et al., 2021). Accordingly, *A. thaliana* plants overexpressing MAPK genes feature a positive regulation on ABA-mediated stomatal closure, thus displaying an increased drought tolerance when compared to wild-type plants (Li et al., 2017). Here, the transcriptional profile of ‘IACSP94-2094’ revealed two particularly induced MAPK kinase kinase (*MAP3K*) isoforms, *MAP3K17* (SCJLRT1006B11.g) and *MAP3K17-like* (SCVPRZ3029A08.g), which are activated by ABA signaling and osmotic stresses in *A. thaliana* (Danquah et al., 2015). Increasing concentrations of cytosolic calcium (Ca^2+^) – a universal secondary messenger - in water-constrained conditions induces signal transduction by Ca^2+^ binding proteins, such as calmodulin-like (CML) proteins, which interact with other dehydration-responsive molecules (Zeng et al., 2015). In fact, CML genes are highly responsive to various stress stimuli in plants (Park et al., 2010; Xu et al., 2011; Vadassery et al., 2012). In rice plants, *CML4* is highly expressed in salt-resistant lines as compared to the susceptible ones under salt stress (Asif et al., 2022). Our expression data revealed an up-regulated *CML4* gene (SCCCLR1001F09.g), particularly in the drought-tolerant genotype. Functional studies overexpressing CML genes have demonstrated improved tolerance to multiple abiotic stresses in plants (Xu et al., 2011; Munir et al., 2016); however, functional assays of *CML4* orthologs are still required.

Drought-induced stomatal closure in plants results in a decreased CO_2_ availability, which impairs photosynthesis (Flexas and Medrano, 2002; Chaves et al., 2009), thereby modulating the expression of several photosynthesis-related genes (Osakabe et al., 2014). This study highlighted a broader set of photosynthetic responsive genes in ‘IACSP94-2094’ under water deficit, as compared to ‘IACSP97-7065’. KOBAS and Mapman analyses showed that drought-stressed ‘IACSP94-2094’ leaves could display more DEGs associated with carbon fixation, carbon metabolism, and photosynthesis – antenna proteins. Beyond the up-regulation of the PSI (*psaF* and *psaO*) and PSII (*psb27* and *psbX*) genes, our results unveiled up-regulated genes involved in the Calvin-Benson cycle, including the large subunit of the ribulose-bisphosphate carboxylase [Rubisco (*rbcL*)], its co-chaperones (*CPN10, CPN20*) and chaperonin (*CPN60*), and a beta-type carbonic anhydrase (*βCA* - SCEQLB1063G04.G). In C4 plants, *β-CA* converts CO_2_ to bicarbonate ( ${\text{HCO}}_{3}^{-}$ ), prompting the carboxylation reaction in mesophyll cells by phosphoenolpyruvate carboxylase (PEPC), which is the basis of the CO_2_ concentration mechanism found in C4 species (Sage et al., 2013; Tofanello et al., 2021). Still, to ensure the maintenance and assembly of functional Rubisco in bundle-sheath cells, the chloroplast chaperonin system is critical, which comprises the enzymes encoded by *CPN10*, *CPN20*, and *CPN60* (Gruber and Feiz, 2018). The up-regulated transcriptional profile of these photosynthesis-related genes encoding dark reaction enzymes in ‘IACSP94-2094’ might be directly related to its higher instantaneous carboxylation efficiency, thus reflecting less sensitivity of photosynthesis and maintenance of WUE under water deficit. Furthermore, ‘IACSP94-2094’ also showed induction of expression of genes encoding light-harvesting chlorophyll a/b-binding proteins (LHCBs), such as *CAB2* (SCJFLR1074A11.G), *CAB4*, and *CP29.1*. These proteins are responsible for the assembly of antenna complexes, which is essential for plants adapting to ever-changing environmental conditions (Jansson, 1994; Jenny et al., 2003; Ganeteg et al., 2004). For instance, the disruption of *LHCB* genes substantially decreases drought tolerance in *A. thaliana* plants, whereas *LHCB6*-overexpressing *A. thaliana* plants display enhanced stomatal sensitivity to ABA (Xu et al., 2012). Collectively, these transcriptional adaptations in the photosynthesis of ‘IACSP94-2094’ suggest a better adaptation of this cultivar to water stress.

Several transcriptional factor (TF) families have been reported to be involved in response to abiotic stresses in sugarcane (Javed et al., 2019). As we reported for a field study (Contiliani et al., 2022), the ‘IACSP94-2094’ sugarcane genome contains a large set of TFs responsive to environmental stresses, which was similarly evidenced at 21 DWI. While the drought-sensitive cultivar showed only ten exclusive TFs as DEGs, ‘IACSP94-2094’ modulated the expression of 18 exclusive TFs. Among the up-regulated TFs in ‘IACSP94-2094’, we found *AP2/DREB RAP2-10*, which is probably involved in the activation of cuticular wax biosynthesis, as reported in drought-stressed *A. thaliana* leaves (Yang et al., 2020). Cuticular wax biosynthesis is related to an enhanced WUE in transgenic poplar plants overexpressing another *AP2* TF – *PeSHN1* (Meng et al., 2019). Moreover, the induced *bHLH035* expression in ‘IACSP94-2094’ might act positively for drought tolerance by regulating photosynthesis, and transpiration, as demonstrated in *A. thaliana* transgenic lines (Dong et al., 2014). Although transpiration and stomatal conductance levels declined in both genotypes under drought, ‘IACSP94-2094’ showed better maintenance of both parameters, with only slight reductions under water deficit. Some other TFs exclusively induced in ‘IACSP94-2094’ have also been shown to be positive regulators for drought tolerance in other plants. For instance, ectopic expression of *A. thaliana DREB1C* improved the growth and yield of drought-stressed rice plants (Ishizaki et al., 2013), and *bZIP23* has been reported to regulate *UDP-glycosyltransferase 2* gene expression in rice, which resulted in enhanced tolerance to salt stress (Wang et al., 2022).

Environmental disturbances, such as drought, prompt the overproduction of reactive oxygen species (ROS) in plants, which operate in signal transduction pathways toward stress responses (Xu et al., 2015; Laxa et al., 2019). The results revealed a genotype-specific up-regulated expression of a respiratory burst oxidase homolog B (*RBOHB*) in ‘IACSP94-2094’, which is a ROS-generating enzyme induced through ABA signaling in drought-stressed plants (Kwak et al., 2003). Previous studies found that ‘IACSP94-2094’ sugarcane plants showed strong antioxidant responses at the transcriptional level during drought stress under field (Contiliani et al., 2022) and greenhouse conditions (Sales et al., 2013). Consistently, ‘IACSP94-2094’ displayed two up-regulated DEGs that are associated with redox homeostasis: H-type thioredoxin (*TRX-H*) and theta glutathione S-transferase (*GSTT*). Additionally, our RT-qPCR analysis disclosed that *GSTU6* transcript accumulates especially in the drought-tolerant genotype. Plant GSTs are well-studied detoxifying enzymes associated with tolerance to various abiotic stresses, including drought, in several crops (Ji et al., 2010; Xu et al., 2018; Zhang et al., 2021). Nevertheless, there is a lack of studies on the overexpression of theta class GST in plants. In a singular study, *AtGSTT1*-overexpressing transplastomic tobacco plants were shown to be tolerant to mannitol-induced osmotic stress and displayed enhanced phototolerance and turgor maintenance under stress (Stavridou et al., 2019). Thioredoxins (TRXs) comprise a large family of crucial plant ROS-detoxifying enzymes (Da Fonseca-Pereira et al., 2019), of which the H-type TRX was reported in sugarcane to interact with *SsNAC23* transcription factor under cold stress (Ditt et al., 2011). Therefore, we suggest that sugarcane *TRX-H* might also respond to drought stress in a genotype-dependent manner, especially in the tolerant genotype. The transcriptional responsiveness of the antioxidant machinery of ‘IACSP94-2094’ supports its strong responsiveness and protective role against drought.

In the absence of transcriptome studies focused on sugarcane WUE under water deficit, a few studies in close related grasses have contributed to elucidate some of the genetic components of the plant responses (Fan et al., 2015; Fracasso et al., 2016; Yuan et al., 2022). Fan et al. (2015) reported 48 genes transcriptionally connected to improved WUE in *Miscanthus lutarioriparius*, which were assigned to photosynthesis, stomatal regulation, and responses to abiotic stresses. Among these genes, our transcriptome analysis of ‘IACSP94-2094’ showed three possible orthologs under drought conditions: thioredoxin-like CXXS1 (*TRX-H*), pyruvate orthophosphate dikinase1 (SCRLCL6031H05.g), and lysine-specific histone demethylase 1 (SCBGLR1023B11.g), whereas in ‘IACSP97-7065’, we found cysteine-rich receptor-like protein kinase 10 (SCSGAD1009D03.g) and soluble starch synthase II-2 (SCSGSB1009B08.b). In sorghum, Fracasso et al. (2016) reported extensive sets of drought-responsive genes involved in photosynthesis, carbon fixation processes, and antioxidant system (Fracasso et al., 2016). Yuan et al. (2022) reported similar pathways for a drought-tolerant broomcorn millet (*Panicum miliaceum* L.) genotype, in which drought-triggered transcriptome was mainly enriched in signal transduction, MAPK signaling, and carbon metabolism pathways (Yuan et al., 2022). As our sugarcane data are in agreement with these reports, we encourage functional genomic studies focusing on the overexpression and/or knockout (*e.g*., conventional transgenesis or CRISPR/Cas) of sugarcane genes underlying these biological pathways.

In summary, the present study disclosed the physiological and transcriptional adaptations of contrasting sugarcane genotypes in coping with drought stress conditions. The drought-tolerant genotype ‘IACSP94-2094’ displays superior WUE under water deficit and rehydration, relying on less sensitivity on reducing carbon assimilation under drought and increasing water use after rehydration. Accordingly, this genotype also shows a stable carboxylation efficiency under water deficit, with higher levels than the drought-sensitive genotype. Similar to a previous microarray study (Contiliani et al., 2022), our RNA-seq data showed that the transcriptional profile of ‘IACSP94-2094’ was more responsive than ‘IACSP97-7065’ to drought, thus pinpointing a higher number of DEGs under water-limiting conditions. Remarkably, most of the candidate genes in ‘IACSP94-2094’ were functionally represented by photosynthesis, transcription factors, signal transduction, solute transport, and redox homeostasis. Based on our data, we conclude that these biological pathways play pivotal roles in drought tolerance and WUE of sugarcane plants, and further functional validation of their candidate genes may unlock novel strategies or markers for breeding programs.

## Data availability statement

The datasets presented in this study can be found in online repositories. The names of the repository/repositories and accession number(s) can be found below: NCBI Sequence Read Archive (SRA) database (BioProjectID: PRJNA882367).

## Author contributions

SC, AF, and RR conceived and supervised the project, designed the experiments, and acquired resources. ML developed the sugarcane genotypes. JN and RR performed the physiological experiments. RM provided expertise in RNA sequencing procedures. JN performed RNA sequencing and RT-qPCR analysis. TP and RR contribute with formal data analysis and text editing. DC curated the RNA-Seq data, conducted functional analyses, prepared all figures and tables, and wrote the entire manuscript. All authors read and approved the final manuscript. All authors contributed to the article.
