# Supplementary figures and images for: Drought-triggered leaf transcriptional responses disclose key molecular pathways underlying leaf water use efficiency in sugarcane (Saccharum spp.)
Source: Front Plant Sci. 2023 May 8;14:1182461. doi: 10.3389/fpls.2023.1182461 (PMC10200899; doi:10.3389/fpls.2023.1182461)

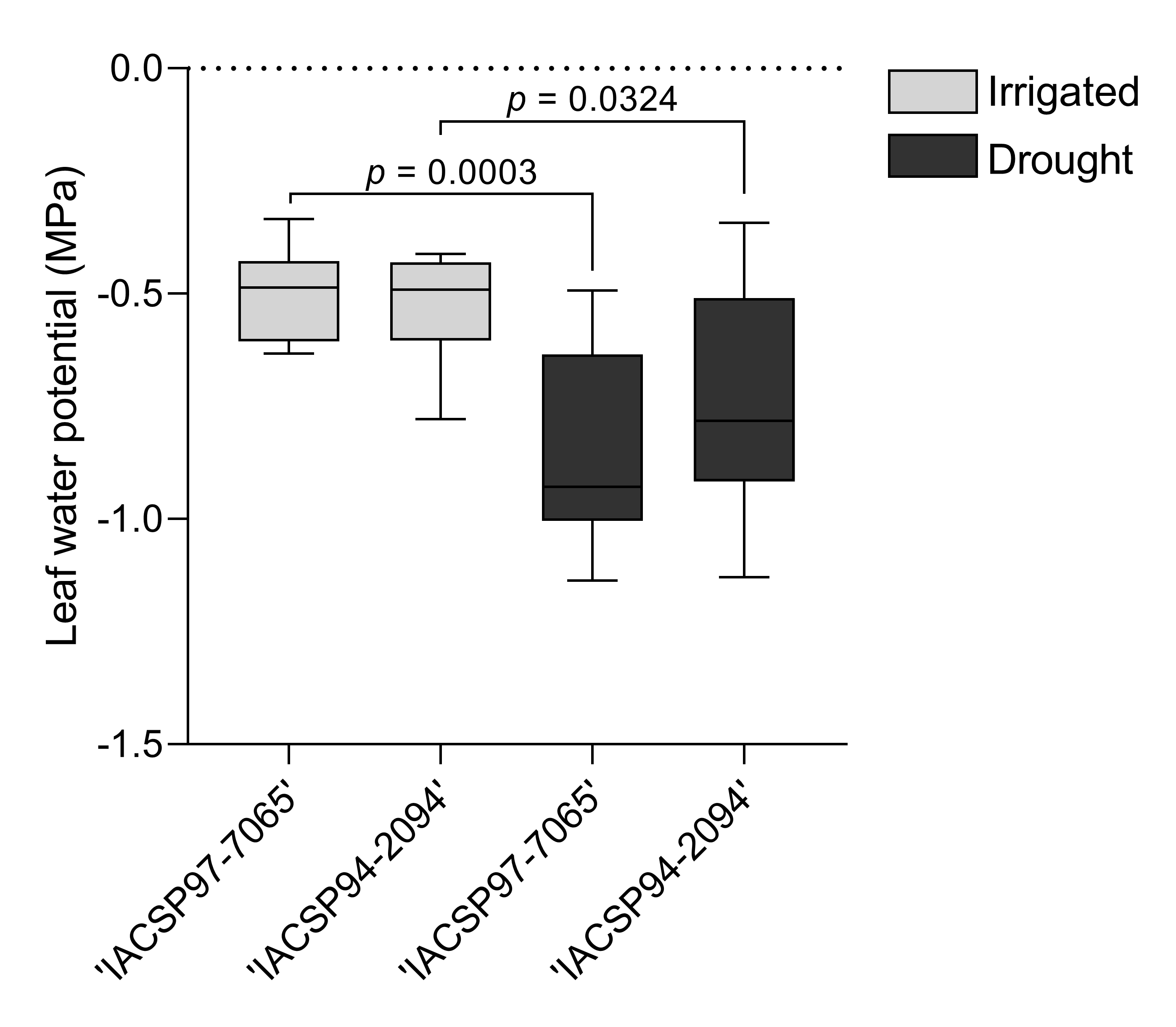

Supplement: Supplementary file 1 [file Image_1.tif]

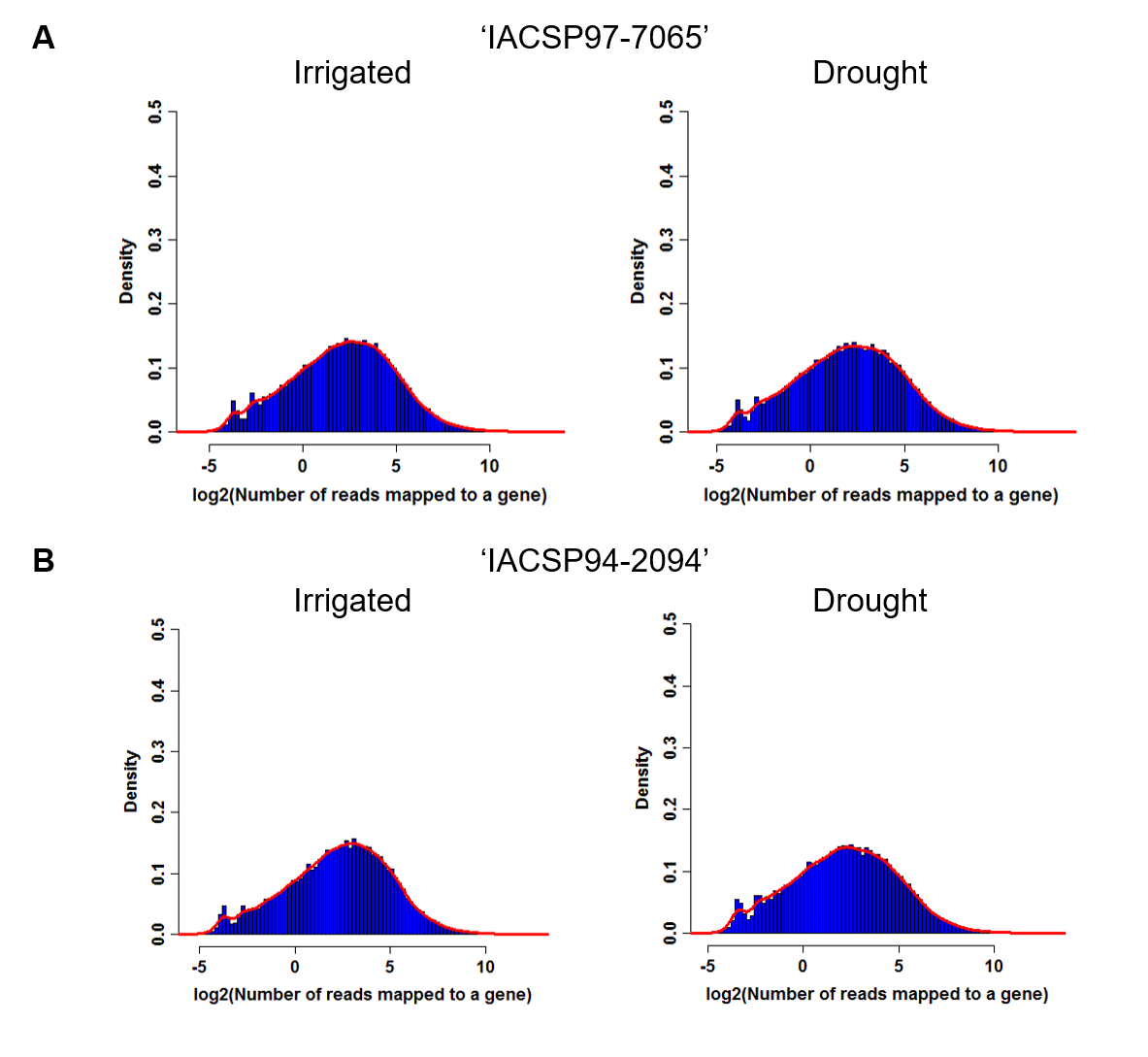

Supplement: Supplementary file 2 [file Image_2.tif]

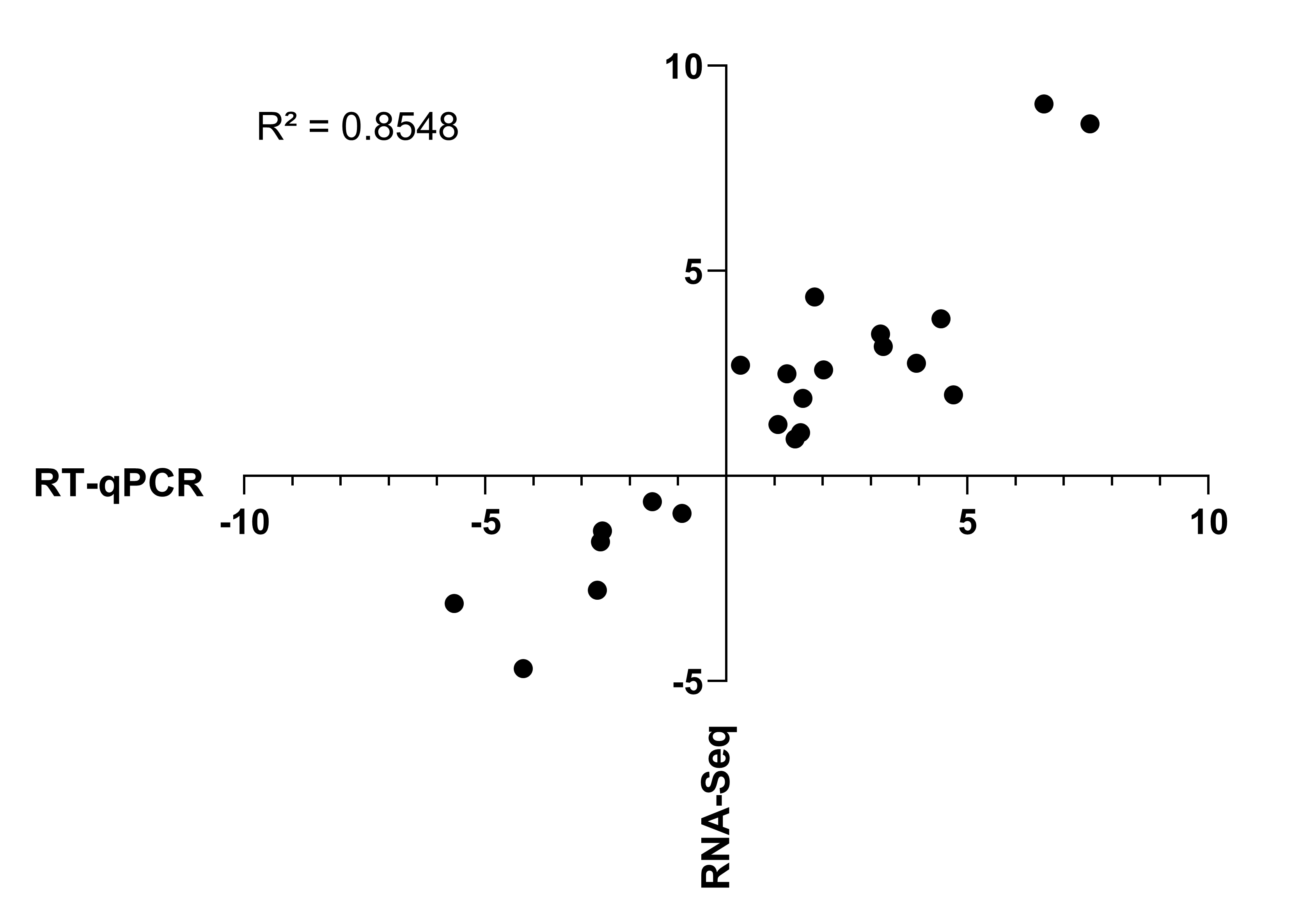

Supplement: Supplementary file 3 [file Image_3.tif]
